# Reflexão sobre Conflitos de Interesses em Diretrizes Médicas

**DOI:** 10.36660/abc.20200104

**Published:** 2020-11-01

**Affiliations:** 1 Instituto do Coração São PauloSP Brasil Instituto do Coração (Incor), São Paulo, SP – Brasil

**Keywords:** Medicina, Profissionalismo, Diretrizes Clínicas, Conflito de Interesses, Fidelidade a Diretrizes, Bioética

A prescrição de um fármaco diretamente ao paciente, a captação do voluntário para uma pesquisa clínica, a palestra em um congresso da especialidade, a participação em um comitê elaborador de uma diretriz clínica, a supervisão em uma visita clínica com residentes, a opinião para os colegas em um cafezinho. Todas essas situações se caracterizam como interação humana, maior ou menor assimetria de conhecimento e influência de múltiplos interesses, em que o eventual privilégio de um pode provocar prejuízo de algum outro.

Dessa maneira, estabelece-se a potencialidade do confronto entre interesses,^1^^,^^2^ o que recebe a denominação de *potencial conflito de interesses*. O uso do termo “potencial” já traz a inquietude de uma possibilidade da condição humana que, contudo, não ocorreu, podendo nunca acontecer ou se materializar em uma recepção imperceptível ou emissão inconsciente.

A areia movediça está na própria essência da medicina, a necessidade de realizar um profissionalismo sob moldura ética, moral e legal. Isso demanda desdobramentos clínicos, técnicos, científicos e atitudinais sob forte indeterminação acerca da beneficência (conceitual) e do benefício (individual), da não maleficência (conceitual) e do não malefício (individual).

As diretrizes clínicas são ilustrativas do tema-dilema. Elas ganharam o *status* de recomendação de primeira ordem; assim, a confiabilidade é avalizada por sociedades de especialidade idôneas e servem de ponto de referência para críticas éticas. Se, por um lado, as diretrizes clínicas visam à excelência estética do T (símbolo da abrangência do saber na barra horizontal e da profundidade na barra vertical) em uma proporcionalidade atualizada de acordo com evidências de pesquisas clínicas, por outro lado, vivências profissionais e opiniões de peso, o cotidiano na beira do leito, destaca a sabedoria dos ajustes individualizados. Assim, a matéria-prima do pavio do potencial conflito de interesses é a evidência científica, mas quem costuma riscar o fósforo é a individualização.

Sobressai na elaboração das diretrizes clínicas a seleção colegiada da dimensão de efeito e da probabilidade de realização de métodos em doenças e circunstâncias. Os membros do comitê de elaboração precisam analisar as evidências de determinações recíprocas entre método e situação clínica.

Inclusões, exclusões e classificações prescritivas devem ser guiadas pela ideia da reciprocidade entre sal e água, ou seja, em qualquer local, a água dissolve o sal e o sal dissolve-se na água, desde que líquida, não cabendo a regra para gelo ou vapor d'água. Ao mesmo tempo, deve-se ter em mente que cada método diagnóstico, terapêutico ou preventivo representa, invariavelmente, um bastão, em que uma extremidade carrega beneficência e a outra, maleficência. Portanto, não existe iatrogenia zero para o paciente, as bulas assim ensinam.

Será que a manifestação de um conflito de interesses por parte do membro do comitê de elaboração de diretriz constitui desejável vacina moral? Creio que não. Uma coisa é a plateia calibrar os poros do seu filtro crítico sobre o que o palestrante afirma, outra coisa são as bases para o leitor de diretrizes clínicas supor vieses.

Do ponto de vista pragmático, não se pode ignorar que os critérios de qualificação da escolha dos especialistas para elaborar uma diretriz superpõem-se aos utilizados pela indústria para a ela se associar de alguma maneira. A ligação acadêmica, a produção científica continuada, o crédito entre os colegas são essências comuns. Em decorrência disso, é alta a chance de se pensar em um nome e colidir com algum potencial conflito de interesses. Posições radicais podem comprometer a seleção, algo como afunilar na direção dos mais inexperientes.

A bioética da beira do leito entende que a manifestação de um conflito de interesses no corpo de uma diretriz tem o único objetivo de afirmar: *dou a minha palavra de honra que tenho tais potenciais teóricos, mas não os realizei*.

É de se supor que coadores de responsabilidade da Sociedade Brasileira de Cardiologia precederam a leitura de uma nova diretriz clínica nos Arquivos Brasileiros de Cardiologia, tais como a decisão sobre a necessidade de atualização/primeira vez, a seleção de nomes, a crítica do elaborado e a aprovação final. Por isso, o foco de confiança e a responsabilidade concentram-se na gestão societária.

As inquietudes da gestão podem ser simplificadas na tríade: não informação, informação enviesada e informação qualificada. A complexidade é que qualquer um desses pode ser objeto do conflito de interesses. Assim, escamotear uma novidade, forçar uma recomendação ou dar ênfase a uma evidência de fato endossável podem embutir interesses pessoais ou de indivíduos ligados.

A bioética da beira do leito prefere o foco na fidelidade à própria consciência no desempenho de funções sujeitas a imperfeições da condição humana.^3^ Evidentemente, fortes associações à indústria devem ser evitadas, dentro do conselho de que não basta ser honesto, é preciso evitar as dúvidas.

Não obstante, é capital considerar que o comportamento dos membros do comitê de elaboração de diretrizes será invariavelmente de responsabilidade para com a função em grupo, de interesse prioritário em prol do coletivo, de rejeição a qualquer expressão de autômato dentro do grupo, de respeito com crítica pelo coordenador-líder, de compasso entre as conclusões de pesquisas e as realidades da beira do leito; enfim, de liberdade e de independência bem sustentadas pela plataforma científica atualizada e validada.

Alguém pode conjecturar que seria ingênuo confiar no imperativo da consciência,^4^ que mesmo um especialista, um professor-doutor, qualquer jurado por Hipócrates e com um compromisso com a sociedade pela posse de um número de CRM, não pode deixar de ser arrastado pela superficialidade de um interesse espúrio quando dele é exigida a profundidade do seu saber e sabedoria. É contraponto válido; porém (sempre tem um porém), quem pode negar que a manifestação do conflito de interesses, não somente impossibilita definir os descontos necessários na recepção, como também não funciona como um “007” moral – licença para conflitar. Há suposições sobre um exagero estratégico, uma tendência ao emissor prover mais vieses para contrabalançar os descontos de recepção provocados pela manifestação dos conflitos de interesses. Por outro lado, pode inibir contraposições temendo enquadramento em realização de conflito de interesse.

Há 26 séculos, Eubulides de Mileto fez a pergunta: *“Em que momento um monte de areia deixa de sê-lo quando se vai removendo grãos, ou um grão se torna um monte pelo acréscimo sucessivo?”* Só haverá resposta de modo autoritário, se alguém estabelecesse um critério com algum tipo de força impositiva. Quanto de flexibilidade pode ser tolerável na opinião de um membro de comitê de diretrizes?

Dada a presunção de honestidade profissional dos sócios, que deve vigorar em uma sociedade de especialidade e a dificuldade de percepção de realizações de conflito de interesses em uma medicina contemporânea plena de indeterminações e de metamorfoses aceleradas, creio que o potencial conflito de interesses é parte indissociável da elaboração de qualquer diretriz clínica, que é impossível qualquer tipo de certeza de ausência da realização. Por isso, uma diretriz não é uma algema, mas uma bússola. Assim, ajustes individuais são bem-vindos.

Por isso, independentemente da manifestação dos interesses de cada membro do comitê de elaboração das diretrizes pertinentes ao documento, proponho uma manifestação ao início de cada diretriz: *A Sociedade Brasileira de Cardiologia, desde a decisão da elaboração até a autorização de publicação, manteve inabalada a confiança na boa-fé dos participantes, a virtude que faz da verdade científica um valor para a condução das relações consigo mesmo e com os colegas e pacientes.*
