# Supplementary material for: Reflexão sobre Conflitos de Interesses em Diretrizes Médicas
Source: Arq Bras Cardiol. 2020 Nov 1;115(5):807–8. [Article in Portuguese] doi: 10.36660/abc.20200104 (PMC8452216; doi:10.36660/abc.20200104)
Supplement: Translation

# Translation: A Reflection on Conflicts of Interest in Medical Guidelines

**Affiliations:** 1 São PauloSP Brazil Instituto do Coração (Incor), São Paulo, SP – Brazil

**Keywords:** Medicine, Professionalism, Guidelines, Clinics, Conflict of Interest, Guideline Adherence, Bioethics

Prescribing a drug directly to the patient, recruiting a volunteer for a clinical research, lecturing at a specialty congress, participating in a committee that elaborates clinical guidelines, supervising a clinical visit with residents, giving your opinion for colleagues over coffee.

All of these situations are characterized by human interaction, greater or lesser asymmetry of knowledge, and multiple interests, where the possible privilege of one may cause damage to another.

Thereby, the potential for confrontation between interests is established,^1^^,^^2^ and this is called a potential conflict of interest. The use of the term “potential” brings about a possibility of the human condition that did not occur, and may never happen or materialize as an imperceptible reception or unconscious emission.

Quicksand is in the very essence of medicine: the need of professionalism under an ethical, moral and legal framework that demands clinical, technical, scientific and attitudinal actions under a strong irresolution about beneficence (conceptual) and benefit (individual), of (conceptual) (non-) maleficence and (individual) (non-) harm.

Clinical guidelines illustrate well the dilemma. They have gained first-rate recommendation status; their reliability is guaranteed by trustworthy specialist societies and serve as a reference for ethical criticism.

If, on the one hand, clinical guidelines aim at the esthetic excellence of the letter T—symbolyzing the comprehensiveness of knowledge in the horizontal bar and its depth in the vertical bar—in a proportion that follows evidence from clinical research, professional experiences and strong opinions, on the other hand, the bedside routine highlights the wisdom of individualized adjustments. The raw material for a potential conflict of interest is scientific evidence, but it is individualization that usually lights the match.

The collegiate selection of the effect dimension and the probability of carrying out methods in diseases and circumstances stand out in the creation of clinical guidelines. The steering committee members need to analyze evidence of reciprocal determinations between method and clinical settings.

Inclusions, exclusions and prescriptive classifications must be guided by the idea of reciprocity of salt and water, that is: no matter the conditions, water dissolves salt and salt dissolves in water—as long as in liquid state, not fitting for ice or water vapor. At the same time, it must be borne in mind that each diagnostic, therapeutic or preventive method invariably represents a stick that carries benefit in one end and harm on the other; there is no zero iatrogenesis for the patient, as the medicines' leaflets teach us.

Does the manifestation of a conflict of interest on the part of a member of a guideline committee constitute a desirable moral vaccine? I do not think so. The audience calibrating the pores of its critical filter on what the speaker says is one thing; the foundation for the reader of clinical guidelines to assume bias is another.

From a pragmatic point of view, one cannot ignore that the qualification criteria for the choice of specialists to develop a guideline overlap with those used by the industry to associate itself with it in some way. Academic liaison, continued scientific production, credit among colleagues are common factors. As a result, the chances of thinking about a name and stumbling with any potential conflict of interest are high. Radical positions can impair selection, narrowing it towards the less experienced.

Bedside bioethics understands that the manifestation of a conflict of interest in a guideline has the sole purpose of stating: “I give my word of honor that I have such theoretical potentials, but I did not put them to practice”.

One can assume that people help accountable at the Brazilian Society of Cardiology preceded the reading of a new clinical guideline in the Brazilian Archives of Cardiology, including the decision on the need for updating/first time, selection of names, criticism and final approval. So, the focus of trust and responsibility is on corporate management.

Management concerns can be simplified in the triad: absent information, biased information, qualified information. Any of these can be object of conflict of interest, hence the complexity. Hiding a novelty, forcing a recommendation or emphasizing endorsable evidence can embody personal interests or those of connected individuals.

Bedside bioethics prefers to focus on fidelity to one's conscience when performing functions subject to the imperfections of human condition.^3^ Of course, strong associations with the industry should be avoided, as only being honest is not enough; one must avoid doubts.

‘Nevertheless, it is essential to consider that the behavior of members of a guideline committee will invariably be responsibility of the group, with priority interest aimed for the collectivity, rejecting any automaton expression within the group, respecting the criticism by the lead coordinator, walking side to side with research findings and bedside reality; to sum up: freedom and independence well supported by the updated and validated scientific platforms.

One can guess that it would be naïve to trust the imperative of conscience^4^ that even an expert, a professor PhD, any juror of Hippocrates, committed to society by the possession of a medical register, cannot help but be dragged by the superficiality of a spurious interest when deep knowledge and wisdom is required of them. It is a valid counterpoint, but—and there is always a but—who would deny that the manifestation of conflict of interests not only does not allow the necessary discounts to be defined at the reception, but also does not function as a moral agent 007, bearing a license to conflict. There are suppositions of strategic exaggeration, a tendency for the issuer to provide more biases to offset the reception discounts caused by conflicts of interest. On the other hand, it can inhibit opposition by the fear that it might lead to a conflict of interest.

Eubulides de Miletus asked this question 26 centuries ago: At what point does a pile of sand cease to exist? When grains are removed? Or do grains become a pile by successive addition? The answer is only possible if we look from an authoritarian point of view, if someone establishes a criterion with some type of imposing force. How much flexibility can be tolerated in a guideline committee member's opinion?

Given the presumption of professional honesty by the partners, which should prevail in a specialty society, and given the difficulty in perceiving conflicts of interest in the contemporary setting of medicine, full of undetermined and accelerated metamorphoses, I believe that a potential conflict of interests is an inseparable part of the elaboration of any clinical guideline, and that any kind of certainty of its absence is impossible. Therefore, a guideline is not a handcuff, but rather a compass. Individual adjustments are welcome.

Therefore, regardless of the expression of interests of each member of the guideline committee pertinent to the document, I propose a manifestation at the beginning of each guideline: since the decision of creation until the authorization of this publication, The Brazilian Society of Cardiology kept the confidence in the good faith of participants, a virtue that makes scientific truth a value underlying the relationships with oneself and with colleagues and patients.
